# Acute Exposure to Commonly Ingested Emulsifiers Alters Intestinal Mucus Structure and Transport Properties

**DOI:** 10.1038/s41598-018-27957-2

**Published:** 2018-07-03

**Authors:** Jaclyn Y. Lock, Taylor L. Carlson, Chia-Ming Wang, Albert Chen, Rebecca L. Carrier

**Affiliations:** 1 Department of Bioengineering, Northeastern University, Boston, Massachusetts 02115 USA; 2 Department of Chemical Engineering, Northeastern University, Boston, Massachusetts 02115 USA

## Abstract

The consumption of generally regarded as safe emulsifiers has increased, and has been associated with an increased prevalence of inflammatory bowel and metabolic diseases, as well as an altered microbiome. The mucus barrier, which selectively controls the transport of particulates and microorganisms to the underlying epithelial layer, has been previously shown to be altered by dietary salts and lipids. However, the potential impact of emulsifiers on the protective mucus barrier, its permeability, and associated structural changes are not clear. In this study, we analyzed changes in the mucus barrier to both passively diffusing nanoparticles and actively swimming *E*. *coli* upon exposure to two emulsifiers, carboxymethylcellulose (CMC) and polysorbate 80 (Tween). When exposed to CMC, mucus pore size decreased, which resulted in significantly slower *E*. *coli* speed and particle diffusion rates through mucus. Tween exposure minimally impacted mucus microstructure and particle diffusion, but increased *E*. *coli* speed in mucus. Moreover, both emulsifiers appeared to alter mucus amount and thickness in rat intestinal tissue and mucus-producing cell cultures. These results indicate that acute exposure to emulsifiers impacts barrier and structural properties of intestinal mucus, modulating interactions between intestinal lumen contents, microbes, and underlying tissue, which may contribute to development of intestinal inflammation.

## Introduction

The gastrointestinal tract is lined with mucus, a continuously renewed and selective barrier regulating particulate and nutrient diffusion, and microbe transport to the underlying epithelial barrier^1–4^. Mucus is a complex hydrogel composed of water, glycoproteins, lipids, proteins, salts, cellular macromolecules (e.g., secretory immunoglobulin A), and cellular debris^1,5^. The major glycoprotein in intestinal mucus is MUC2, which is a large gel-forming mucin (molecular mass >500 kDa). Both non-covalent (e.g., hydrophobic and ionic) and covalent (i.e. disulfide bond) mucin-mucin interactions^5^, as well as interactions with other mucus components (e.g., covalent cross-links with proteins such as immunoglobulin Fc gamma binding protein^6^) result in the mesh-like structure that constitutes the mucus gel. MUC2 contains a polypeptide backbone and oligosaccharide side chains that contain varying amounts of five major sugars, galactose, fucose, *N*-acetylgalactosamine, *N*-acetylglucosamine, and sialic acid. These mucin sugars serve as binding sites for pathogenic and commensal microbes, thus preventing microbes from adhering to the epithelial cells^7^.

It has been demonstrated that mucus barrier properties can be altered upon exposure to stimuli presented by intestinal contents. For example, food-associated stimuli, including lipids and calcium, were found to enhance mucus barrier properties and decrease the diffusion of particulates in intestinal mucus, potentially by altering intermolecular (e.g., mucin-mucin) interactions within the mucus gel^8^. Similarly, small angle neutron studies demonstrated that polyphenols (e.g., epigallocatechin gallate and epigallocatechin) derived from green and black teas cross-linked mucin fibers, resulting in an increase in viscosity and elastic shear modulus of the mucus gel^9^. Fruit and grain dietary fiber polysaccharides were found to bind with mucins by mucoadhesive molecular interactions and polymer interpenetration, which increased the viscosity of a 1% w/w porcine intestinal mucin solution^10^. These studies have shown that the mucus barrier can be altered by different stimuli associated with materials we commonly ingest.

Penetration of commensal and pathogenic bacteria through mucus, associated adherence to and invasion of the epithelium, as well as reduced thickness of the mucus barrier have been associated with mucosal inflammation, such as occurs in inflammatory bowel disease (IBD)^11–13^, a disease affecting approximately 1.6 million Americans^14^. These observations suggest that an altered mucus barrier may contribute to the onset of intestinal inflammation and certain disease states^15^. In a recent study by Chassaing *et al*., two emulsifiers present in processed foods, carboxymethylcellulose (CMC) and polysorbate 80 (Tween), were found to induce inflammation and increase the proximity of microbes to the epithelia *in vivo*^16^. When added to drinking water (1% wt/vol) of wild-type mice for 12 weeks, CMC and Tween resulted in metabolic syndrome and increased secretion of pro-inflammatory cytokines. Microbes were found ~10 μm from the colonic epithelium in both CMC and Tween-treated animals, while they were ~25 μm from the epithelium in healthy mice. The encroachment of microbes was associated with a decreased mucus thickness, but not a decreased expression of MUC2, the main secreted gel-forming mucin in the intestine. Exposure to CMC and Tween also reduced intestinal microbiota diversity and increased the number of mucolytic microbes, such as *Ruminococcus gnavus*. Interestingly, emulsifier-exposed microbiota transplanted into germ-free mice resulted in low-grade intestinal inflammation suggesting direct exposure to emulsifiers was not necessary for emulsifier-induced inflammation^17^. Moreover, Singh *et al*. showed that a single dose of 1% Tween per kg (body weight) per day in mice for 40 days resulted in liver disease, where hepatocytes had large mitochondria indicating increased oxidative stress compared to control mice^18^. The amount of emulsifiers in processed foods (e.g., ice cream ~0.5% emulsifiers^19^, fruit juice ~0.4% emulsifiers^20^, canned soups ~0.25–1% emulsifiers^20^) as well as drug and vitamin products have increased, and the global market for emulsifiers is expected to reach 2.9 billion dollars by 2018^21^. While certain emulsifiers have been classified by the United States Food and Drug Administration (FDA) as generally regarded as safe (GRAS), it is crucial to understand if and how the increased consumption of emulsifiers may contribute to the rise in obesity and other intestinal inflammatory diseases^13,22^.

Herein, we test the hypothesis that acute exposure to CMC and Tween directly impacts mucus barrier properties, to determine if such changes in this crucial barrier could contribute to the ultimate development of inflammation and metabolic syndrome. We visually observed changes to the mucus gel upon emulsifier exposure using confocal microscopy. To probe mucus barrier properties in the presence of emulsifiers, fluorescent nanoparticles and microbes (*Escherichia coli* (*E*. *coli*)) were tracked within mucus using multiple particle tracking (MPT)^23–26^. Given the common consumption of processed foods containing emulsifiers, understanding the impact of these substances on mucus barrier properties could shed important light on mechanisms by which what we eat contributes to bacterial infection and intestinal inflammation.

## Experimental Section

### Intestinal mucus collection

Native porcine small intestinal mucus was harvested and rinsed with cold water to remove bulk material at local abattoir (Research 87 Inc., Boylston MA). The mucus layer was gently scraped using a metal spatula, and then stored in microcentrifuge tubes at −80 °C. Frozen mucus was thawed for 30 minutes at room temperature prior to use.

### Nanoparticle preparation and characterization

Carboxyl- and amine- modified yellow-green 200 nm diameter fluorescent nanoparticles (FluoSpheres®, Life Technologies) were used to probe the mucus barrier. Particle surfaces were functionalized with polyethylene glycol (PEG) using 1-ethyl-3-(3-dimethylaminopropyl)-carbodiimide (EDC, Sigma) cross-linking chemistry to covalently conjugate 2000 molecular weight (MW) amine-terminated PEG (PEG-NH_4_, Laysan Bio, Inc.) to carboxyl- modified particles. Briefly, the carboxyl- modified particles were diluted in 50 mM 2-(N-morpholino) ethanesulfonic acid (MES buffer, pH 6.5, Sigma), then 20 mg PEG-NH_4_ and 10 mg EDC were added to the particle solution and mixed for 2 hrs at room temperature. To quench the reaction, 100 mM glycine (Sigma) was added and mixed for 30 mins. The particle solution was dialyzed overnight using Slide-A-Lyzer® Mini Dialysis Devices, 10 kDa cut off (ThermoFisher Scientific). To determine particle size and zeta potential, a dynamic light scattering and zeta-sizer instrument (NanoBrook 90Plus PALS, Brookhaven) was utilized.

### Nanoparticle and mucus sample preparation

Transport properties of 200 nm fluorescent particles with carboxyl, amine, and PEG surface functionalization were probed using multiple particle tracking technique. The different surface functionalities were used to study the effect of charge on particle-mucus interactions. To prepare particle suspensions, the particles were diluted to a final concentration of 0.0025% wt/vol in the presence of 1% carboxymethylcellulose (CMC ~250,000 MW), 1% Polysorbate 80 (Tween), or maleate buffer (MB) as control. MB solution was composed of 100 mM Tris-maleate, 65 mM sodium chloride, 10 mM calcium chloride, 3 mM sodium azide, and 4 mM sodium hydroxide, at pH 6.5. Concentrations of CMC and Tween were chosen to be less than or equal to limits considered by the FDA to be safe^27^.

Thawed porcine intestinal mucus (150 µL) was added to a Lab-Tek^®^ Chamber Slide™ (Nunc™). Particle suspensions (7.5 µL, carboxyl-, amine-, or PEG- modified particles) in 1% CMC, 1% Tween 80, or MB (control) were vortexed and added drop-wise with minimal perturbation to the mucus surface. While the concentration of emulsifiers added to mucus (1%) may represent the upper limit approved by the FDA, the final concentration of emulsifiers in mucus (~0.048%) is significantly diluted, reflecting dilution by food matrix or lumen contents. The sample was incubated in a dark, humidified chamber for 2 hrs at room temperature prior to imaging. During the incubation, particles diffused through mucus without any additional mixing.

### *E*. *coli* preparation

*Escherichia coli* (*E*. *coli* MG1655) was used as a model microbe. *E*. *coli* was transformed with a green fluorescent protein (GFP) plasmid (p-mut2-GRP, gift from Marcia Goldberg) using standard heat-shock protocol to obtain fluorescent *E*. *coli* for tracking. Briefly, a 16 hr overnight culture of *E*. *coli* was diluted 1:100 in luria bertani (LB) broth and cultured for 2 hrs. The culture was centrifuged at 5000 rpm, 4 °C for 10 mins. The pellet was re-suspended in cold 100 mM calcium chloride (CaCl_2_) solution for 30 mins at 4 °C. *E*. *coli* centrifuged and re-suspended in 2 ml of CaCl_2_. GFP-containing plasmid was added to 150 μl *E*. *coli* in CaCl_2_ and subjected to heat shock in a 42 °C water bath for 45 secs. The transformed cells were diluted in 1 mL LB broth and plated on ampicillin-containing LB agar. After overnight culture, transformed fluorescent *E*. *coli* were selected, and frozen cultures were prepared in 20% glycerol solution and stored at −80 °C.

The *E*. *coli* from glycerol stocks were cultured in LB broth containing ampicillin overnight (~16 hrs) at 37 °C and 220 rpm. *E*. *coli* (~10^6^ cells/mL) was mixed with test media: 10 μl *E*. *coli* + 5 μl test media (1% CMC, 1% Tween, or MB without sodium azide) and incubated for 5 mins. Then, 10 μl of *E*. *coli* in test media was dosed to 200 μl porcine intestinal mucus or MB and imaged. *E*. *coli* were tracked in both mucus and MB to elucidate the impact of emulsifiers on *E*. *coli* trajectories.

### Multiple particle tracking (MPT)

Particle and *E*. *coli* videos were obtained using an X-Cite 120 fluorescence illumination system and Olympus DP70 digital color camera attached to an inverted Olympus IX51 microscope. Briefly, 20 sec trajectory videos were recorded with frame rate of 30 frames per second. Tracking videos were taken in areas close to droplets, and thus exposed to emulsifiers, but not within the droplet.

#### Analysis of particle diffusion

Particle trajectories were analyzed using custom MATLAB software previously developed^28^ to calculate mean-squared displacement (<MSD>) and effective diffusivity (*D*_*eff*_).

1 $${\rm{MSD}}={[{\rm{x}}({\rm{t}}+{\rm{\tau }})-{\rm{x}}({\rm{t}})]}^{2}+{[{\rm{y}}({\rm{t}}+{\rm{\tau }})-{\rm{y}}({\rm{t}})]}^{2}\,$$

2 $${D}_{eff}=\frac{{\rm{MSD}}}{4{\rm{\tau }}}\,$$

where, x(t) and y(t) represent the particle coordinates at a given time and τ is the time scale. *D*_*eff*_ is used to characterize the diffusion of particles through mucus, a porous medium.

Anomalous diffusion of particle trajectories was characterized by fitting the MSD vs. time scale log-log plot with the following equation:

3 $$MSD=4{D}_{0}{{\rm{\tau }}}^{\alpha }$$

where D_0_ is the time-independent diffusion coefficient, τ is time scale, and α is the anomalous exponent, a parameter used to describe particle motion type. Particles were categorized as immobile (0 < α < 0.2), subdiffusive (0.2 < α < 0.9), diffusive (0.9 < α < 1.0), or uninhibited (1.0 < α), using the slopes of MSD vs. time scale log-log plots^23,29,30^. Trajectories of at least 300 particles were analyzed for each experiment, and 3 separate experiments were performed to account for mucus variability.

#### Microrheological characterization of mucus gel

The frequency dependent local elastic (G′) and viscous (G″) moduli were calculated from ensemble average MSD (<MSD>) of neutral PEG-modified particles in mucus exposed to emulsifiers or MB Control^31–35^. Briefly the viscoelastic spectrum, G(s), is calculated as:

4 $$G(s)=\frac{{k}_{B}T}{\pi as\langle {\rm{\Delta }}{r}^{2}(s)\rangle }$$

where, k_B_ is the Boltzmann constant, T is the absolute temperature, a is the particle radius, s is the Laplace frequency, and $\langle {\rm{\Delta }}{r}^{2}(s)\rangle$ is the unilateral Laplace transform of $\langle {\rm{\Delta }}{r}^{2}(\tau )\rangle =\langle MSD\rangle$. The Fourier transform equivalent of G(s), the complex shear modulus G*(ω), is used to calculate the elastic and viscous moduli using the following equations,

5 $$G^{\prime} (\omega )=|{G}^{\ast }(\omega )|\cos (\pi \alpha (\omega )/2)$$

6 $$G^{\prime\prime} (\omega )=|{G}^{\ast }(\omega )|\sin (\pi \alpha (\omega )/2)$$

7 $$\alpha (\omega )\equiv {\frac{d\mathrm{ln}\langle {\rm{\Delta }}{r}^{2}(t)\rangle }{d\mathrm{ln}t}|}_{t=1/\omega }$$

The phase angle, δ, is calculated using the following equation,

8 $$\tan \,\delta (\omega )=\frac{G\text{'}\text{'}}{G\text{'}}$$

where, δ = 0° or 90° corresponds to a purely viscous liquid or elastic solid, respectively.

#### Analysis of *E*. *coli* transport

*E*. *coli* movement through the mucus gel was tracked using the MPT algorithm mentioned previously. *E*. *coli* speed was calculated from individual trajectories:

9 $$Speed=\frac{\sqrt{{[x(t+\tau )-x(t)]}^{2}+{[y(t+\tau )-y(t)]}^{2}}}{\tau }$$

where, x(t) and y(t) represent the *E*. *coli* coordinates at a given time, t, and τ is the time scale (1 sec). Individual *E*. *coli* traces were visualized using ImageJ Mosaic plugin^36^.

### Mucus structure and pore size

Mucus samples for microscopy studies were prepared in a similar manner as samples used for tracking experiments. Mucus was stained for 15 mins with 5 μL of lectin from *Ulex europaeus* conjugated with TRITC (Sigma) to visualize fluorescent particle distribution. Confocal z-stacks were collected with Zeiss LSM710 microscope.

Mucus (150 μl) samples for electron microscopy were exposed to MB, CMC, or Tween (7.5 μl) for 2 hrs; another mucus sample was exposed to MB, CMC, or Tween overnight in closed vials on an orbital shaker at 4 °C to allow uniform distribution of emulsifiers in mucus. Mucus was fixed overnight at 4 °C with Carnoy’s solution, which consists of 60% ethanol, 30% chloroform, and 10% glacial acetic acid. Samples were dried using critical point drying technique with a Samdri^®^-PVT-3D drier at 40 °C and 1200 psi, and sputter-coated with 10 nm thick layer of gold using Cressington Sputter Coater 208HR. Scanning electron micrographs were taken using a Hitachi S-4800 field emission scanning electron microscope operated at 2 kV. MATLAB software was then used to identify and quantify feret diameter of mucus pores.

### Emulsifier addition to *in vitro* cell culture

Human intestinal carcinoma epithelial (Caco-2 clone C2BBe1) and mucus-producing (HT29-MTX) cells were cultured in Dulbecco’s Modified Eagle’s Medium supplemented with 10% heat-inactivated fetal bovine serum (Atlanta Biologics), 1% GlutaMax™, and 1% Penicillin/Streptomycin. Caco-2 (passage 55–65) and HT29-MTX (passage 35–45) cells were seeded in 9:1 ratio on tissue culture treated 24 well plates at a density of 10^5^ cells/cm^2^. After 3 weeks, cells were exposed to phosphate buffered saline (PBS) control, 1% CMC in PBS, or 0.5% Tween in PBS for 1 hr at 37 °C. Tween concentration was reduced to 0.5% to minimize cytotoxicity since exposure to 1% Tween for 1 hr resulted in cell death. After 1 hr exposure, cells were washed with phosphate buffered saline and stained with LIVE/DEAD™ solution (Molecular Probes™). The mucus layer was also stained for 1 hr at 37 °C with 1 mg/mL wheat germ agglutinin (WGA, Molecular Probes™) for sialic acid and N-acetylglucosaminyl, as well as 1 mg/mL lectin from *Ulex europaeus* (Sigma) for L-fucose. Confocal z-stacks of the mucus layer were obtained at 10× with the same imaging parameters used for each sample from three separate experiments. ImageJ was used to quantify intensity of fucose and sialic acid stains in the maximum intensity z-stack projection. Average values ± standard deviation are reported.

### *In vivo* intestinal exposure to emulsifiers

All methods were conducted in accordance with approved guidelines and regulations. Animal experiments were approved by Institutional Animal Care and Use Committee (IACUC) at Northeastern University under IACUC protocol #15-0316 R. Male Wistar rats, 7–8 weeks old, 200–250 g, were fasted ~16 hrs overnight on a wire-bottom cage to prevent coprophagy. Rats had free access to water during fasting period. Rats were placed on a warm heating pad and anesthetized using 2.5% isoflurane gas. While under anesthesia, the abdominal cavity was exposed with a 3 cm incision approximately 2 cm below the diaphragm. Intestinal loops (~1.5 cm of ileum adjacent to the cecum) were sutured, and a 30 gauge needle was used to inject 25 μL of 1% CMC, 1% Tween, or MB. The intestine was gently placed back in the abdominal cavity for 30 mins. While under anesthesia, the rat was euthanized using thoracotomy followed by exsanguination by cardiac puncture. Small intestinal loops were quickly excised and fixed in Carnoy’s solution overnight at 4 °C. Samples were embedded in paraffin, and ~16 μm thick histological samples were stained with alcian blue, Periodic Acid-Schiff, and hematoxylin.

### Statistical analysis

Particle and *E*. *coli* tracking experiments were completed in triplicate, where at least 300 particles or *E*. *coli* in total were analyzed for each experiment. Structural analyses (i.e., scanning electron and confocal microscopy, and histology) were completed in three separate experiments using mucus collected from three separate animals to account for mucus and tissue variability. All data are presented as a mean with standard error of the mean (SEM). ANOVA was used to determine significance with α = 0.05.

## Results

### Intestinal mucus structural changes

**Figure 1:**
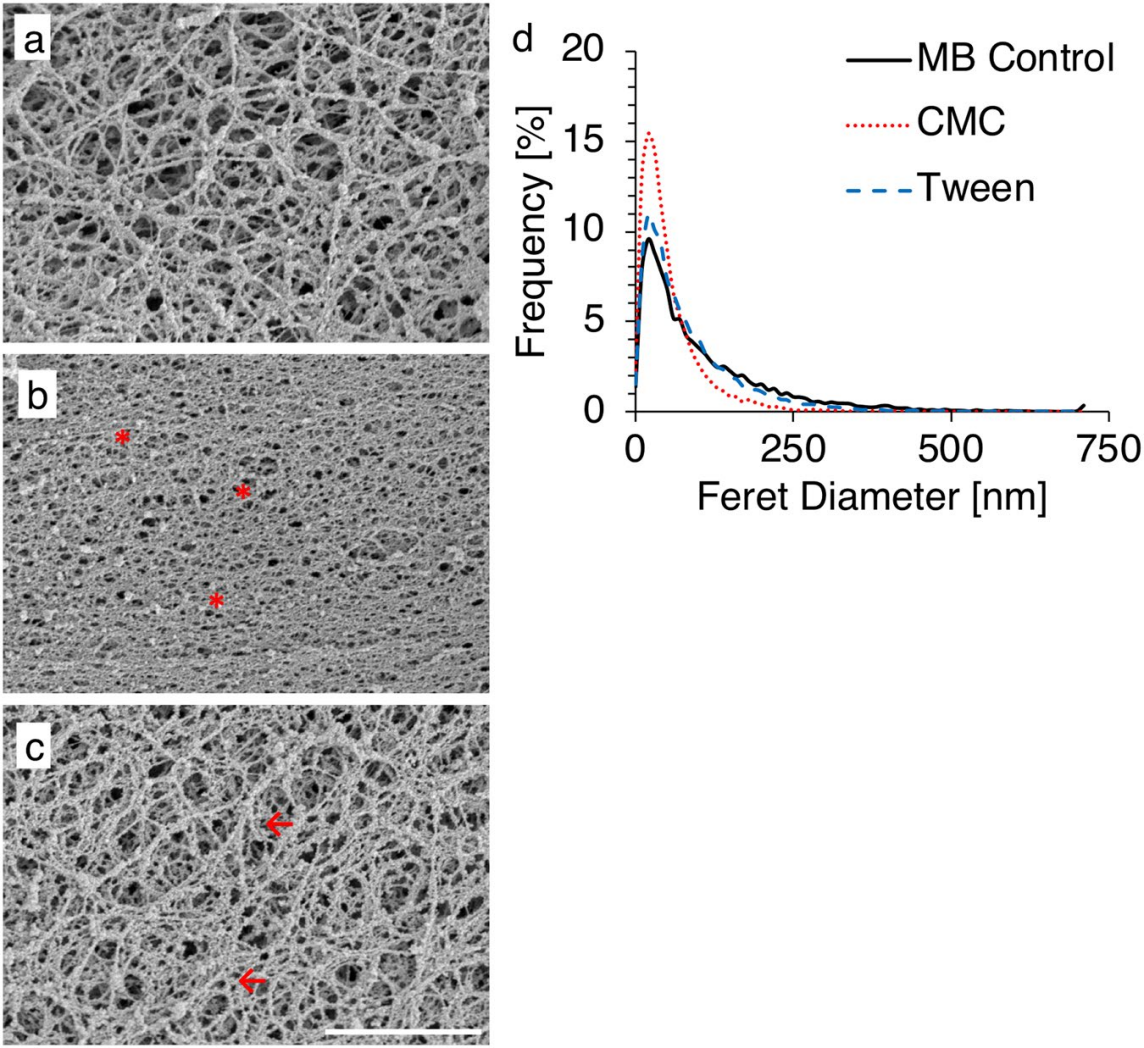
Scanning electron micrographs of mucus exposed for 2 hrs to MB Control (**a**), CMC (**b**), or Tween (**c**). Smaller pores (*) were evident in mucus exposed to CMC, while exposure to Tween appeared to result in clumped fibers (←). Scale bar: 2 μm. (**d**) Frequency distribution of mucus pore feret diameter as quantified in the presence of MB Control, CMC, or Tween.

Exposure to emulsifiers impacted the mesh-like structure of *ex vivo* porcine intestinal mucus, as visualized with scanning electron microscopy (Fig. 1). For all samples, the pore diameter ranged from 10–700 nm (Fig. 1d). The median pore size for mucus was 109.45, 59.3, and 88.24 nm for mucus exposed to MB, CMC, and Tween, respectively. When exposed to CMC, the fibrous mesh appears to be more compacted (relative to the MB control), with a larger percentage of small pores. In mucus exposed to Tween, fibers appeared to be clumped together and pores were slightly smaller than mucus exposed to MB. To ensure uniform distribution of test solutions (MB, CMC, or Tween) in mucus, samples were also mixed overnight. Results showed that mucus exposed to MB, CMC, or Tween had similar structure when incubated for 2 hrs (Fig. 1), or mixed overnight (Supplementary Fig. S1).

**Figure 2:**
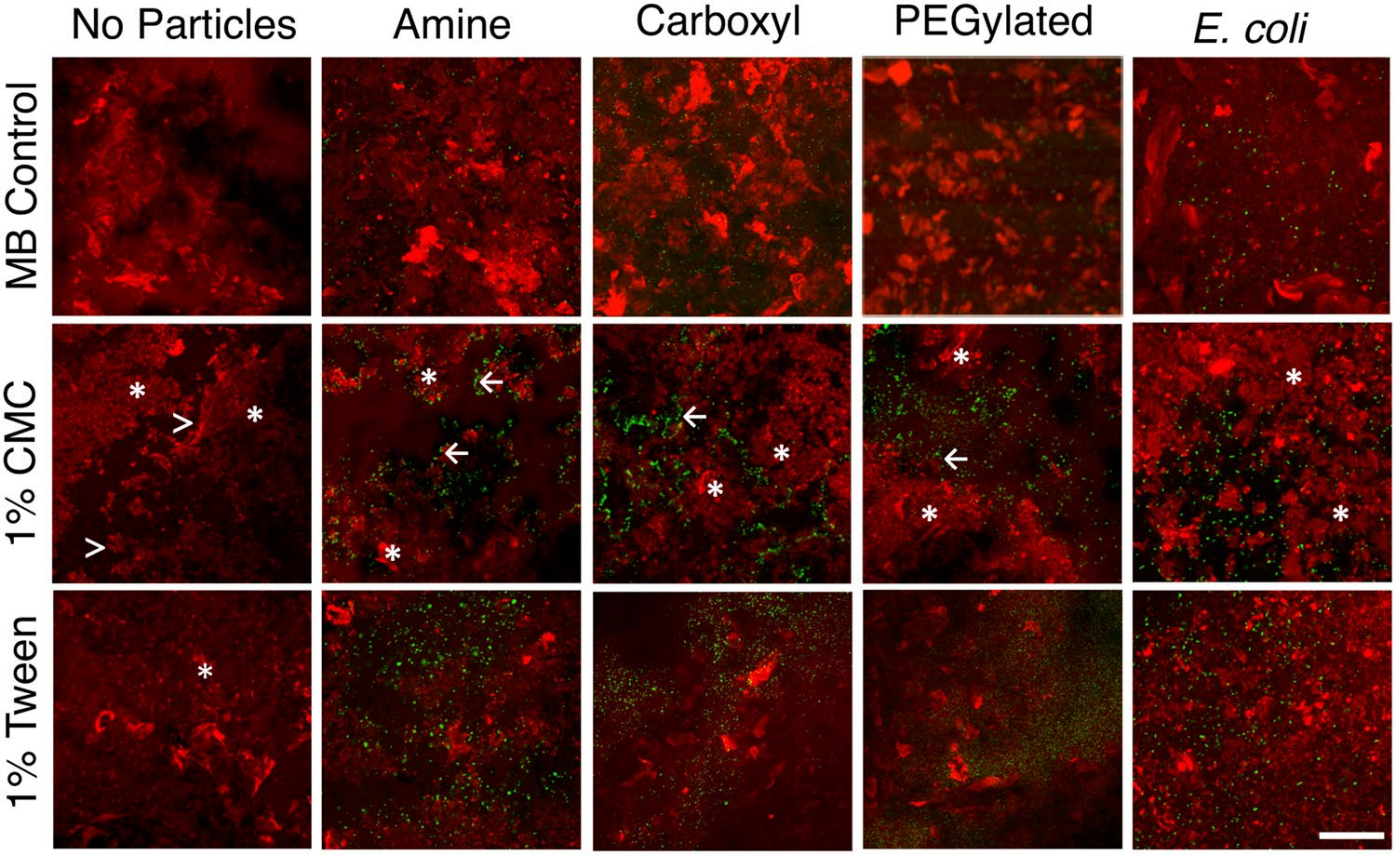
Particles (Amine, Carboxyl, PEG) or *E*. *coli* were mixed with MB Control, CMC, or Tween and added to mucus stained with lectin from *Ulex europaeus*. Mucus exposed to CMC had punctate lectin staining (*), defined edges of lectin-stained regions (>), and agglomerated particles (←). Scale bar: 100 μm.

Exposure to CMC resulted in regions of mucus that were not penetrable by particles, whereas particles were relatively evenly distributed in mucus exposed to Tween or MB (Fig. 2). Particle distribution was visualized by staining mucus with lectin from *Ulex europaeus* and collecting confocal microscopy z-stacks. Exposure to CMC resulted in a more pronounced punctate lectin staining pattern, with defined edges, suggesting possible compaction of the mucus gel. Amine- and carboxyl- modified particles accumulated along these defined edges, rather than being uniformly distributed throughout lectin-stained regions, suggesting limited ability to penetrate these compacted regions of the mucus gel. PEG- modified particles and *E*. *coli* were also found along the defined edges and in areas with low levels of lectin staining. Tween resulted in some punctate lectin staining, but structure as reflected in staining patterns was overall more similar to MB, with particles, regardless of surface functionality, and *E*. *coli* distributed throughout the mucus gel.

Macroscopic structural changes to the mucus gel were assessed by visually observing samples following exposure to MB, CMC, and Tween (Supplementary Fig. S2). Immediately after adding MB, CMC, or Tween to mucus, droplets in the mucus gel were observed. After 2 hrs, MB and Tween exposed mucus samples were similar and appeared to be homogeneous, indicating that the solutions diffused into the mucus. On the contrary, the location of droplets was still apparent in CMC-exposed mucus, which may be due to a compacted mucus structure adjacent to added drops following CMC exposure.

### Particle diffusion in the presence of emulsifiers

**Figure 3:**
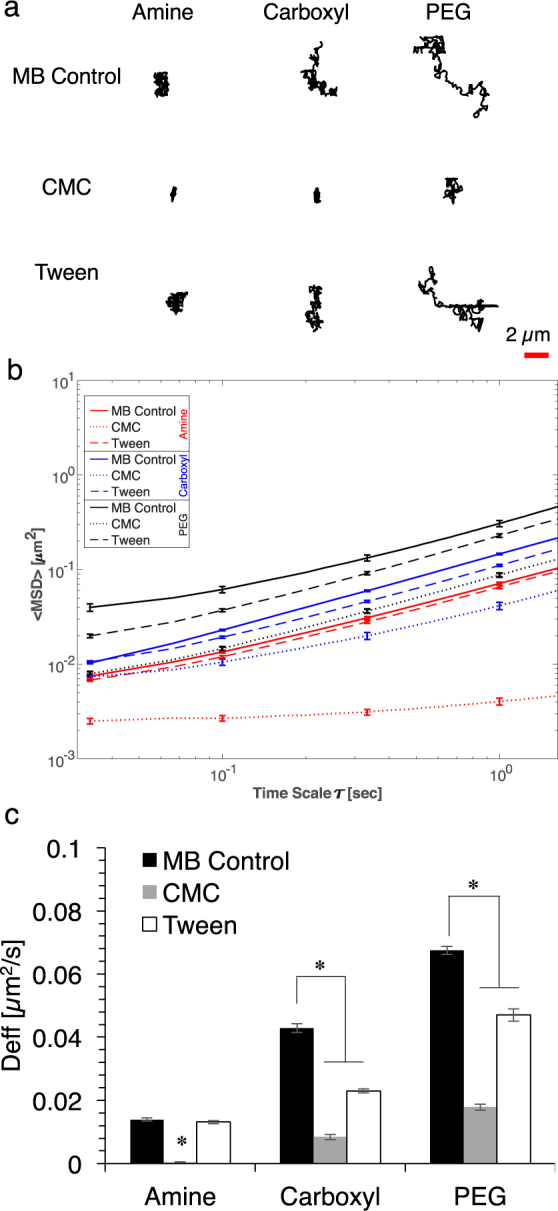
(**a**) Representative trajectories of Amine, Carboxyl, and PEG particles dosed in MB Control, CMC, and Tween to mucus. Scale bar: 2 µm. (**b**) Mean square displacements (<MSD>) as a function of time scale for particles dosed with MB Control, CMC, and Tween. Error bars represent SEM. (**c**) Effective diffusivity (*D*_*eff*_) of particles dosed with MB Control, CMC, and Tween at time scale of τ = 3 sec. Error bars represent SEM and ANOVA was used to determine significance, where * indicates *p* < 0.05 compared to respective control.

Exposure to both emulsifiers modified the mucus barrier, hindering the diffusive motion of particles within mucus (Fig. 3). To determine how the emulsifiers alter the local microenvironment, multiple particle tracking (MPT) technique was utilized to track the diffusion of amine-, carboxyl-, and PEG- modified 200 nm fluorescent nanoparticles in *ex vivo* porcine intestinal mucus. Particle trajectories indicated that diffusion was impacted by particle surface charge, where neutral PEG- (0 ± 5 mV) modified particles diffused over the largest area, negatively-charged carboxyl- (−50 ± 5 mV) modified particles diffused over a smaller area compared to PEG- modified particles, and positively-charged amine- (+28 ± 2 mV) modified particles had the most confined movement. The uncharged PEG-functionalized particles have been characterized as stealth or non-interacting particles that can diffuse freely through pores in the mucus gel^37^. As previously observed, negatively-charged carboxyl particles^8^, diffused more slowly through mucus as compared to the PEG particles, potentially due to electrostatic repulsive forces with the negatively-charged mucin fibers. Conversely, positively-charged amine particles displayed the lowest diffusion rates through mucus, likely due to electrostatic attraction to negatively-charged mucin fibers^38–40^.

Dosing particles to mucus in the presence of CMC resulted in significantly hindered particle trajectories compared to MB for all particle types. When dosed with Tween, carboxyl- and PEG- modified particle trajectories were confined, whereas amine particle trajectories were not affected (Fig. 3a). To quantify these changes in particle diffusion, mean squared displacement (<MSD>) was calculated for at least 300 particle trajectories (Fig. 3b). CMC significantly reduced the <MSD> of all particle types, whereas Tween only significantly reduced <MSD> for carboxyl- and PEG- modified particles.

**Figure 4:**
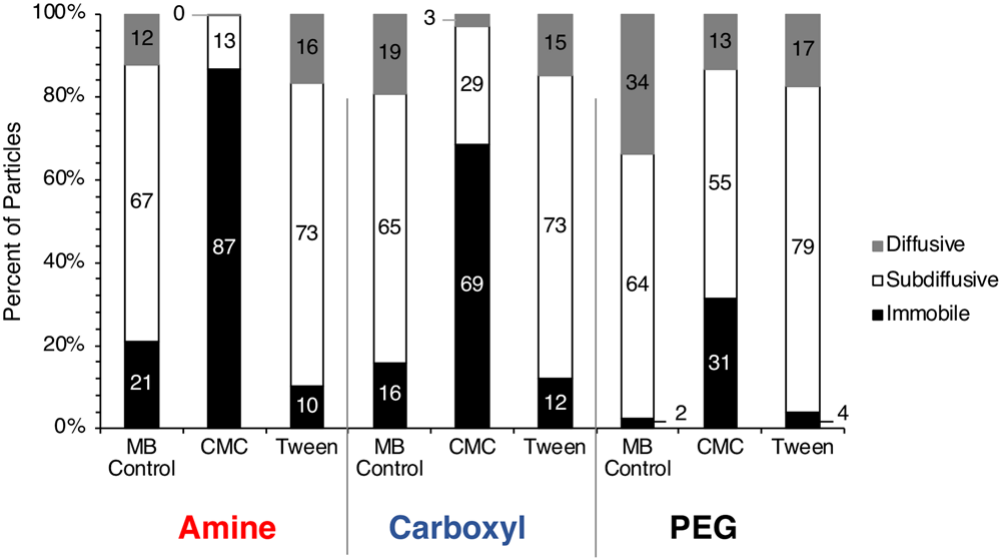
Particle motion type was characterized as immobile (α: 0–0.2), subdiffusive (α: 0.2–0.8), or diffusive (α: 0.8–1.0) by fitting the log-log MSD plot to find the anomalous exponent, α.

At a time-scale of 3 sec, CMC significantly reduced the effective diffusivity (*D*_*eff*_) of amine-, carboxyl-, and PEG- modified particles approximately 30-, 5-, and 4- times, respectively, compared to MB (Fig. 3c). Tween had no impact on *D*_*eff*_ of amine- modified particles, but decreased that of carboxyl- and PEG- modified particles by 2-, and 1.5- times, respectively, compared to MB. CMC resulted in a decrease in subdiffusive (0.2 < α < 0.8) and an increase in immobile (0 < α < 0.2) particles, whereas Tween resulted in a decrease in immobile and an increase in subdiffusive particles (Fig. 4). When compared to MB, average α values were reduced when dosed with CMC from 0.83 to 0.32, 0.86 to 0.76, and 0.90 to 0.85 for amine-, carboxyl-, and PEG- modified particles, respectively, thus indicating that CMC hindered particle diffusion through mucus. In the presence of Tween, average α values were 0.85, 0.86, 0.87 for amine-, carboxyl-, and PEG- modified particles, respectively. These α values were similar to those of MB, showing that Tween did not have an effect on the nature of average bulk diffusion.

### Microrheological analysis

**Figure 5:**
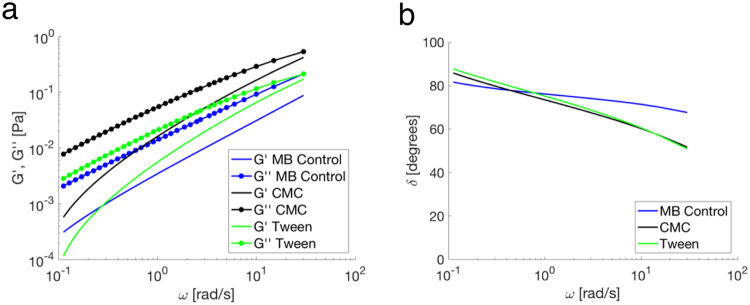
Microrheology of mucus samples. (**a**) Linear viscoelastic moduli and (**b**) phase angle (δ) at different frequencies obtained from microrheological analysis of PEG- modified particles in mucus exposed to MB Control, CMC, or Tween.

Emulsifiers increased the viscoelastic moduli of mucus, where exposure to CMC resulted in the highest elastic (G′) and viscous (G″) moduli values (Fig. 5a). For all samples, G″ was dominant over G′, indicating a liquid-like behavior. The phase angle, δ, was also quantified to determine the state of the mucus gel, where δ = 0° or 90° corresponds to a purely viscous liquid or elastic solid, respectively. For all samples, the phase angle was between 45°–90° indicating mucus samples exposed to MB Control, CMC, or Tween were characterized as a viscoelastic liquid. At higher frequencies (>1 rad/s), the phase angle was lower for CMC and Tween compared to MB Control, indicating that emulsifiers altered the properties of the mucus gel resulting in a more solid-like behavior.

### *E*. *coli* mobility

**Figure 6:**
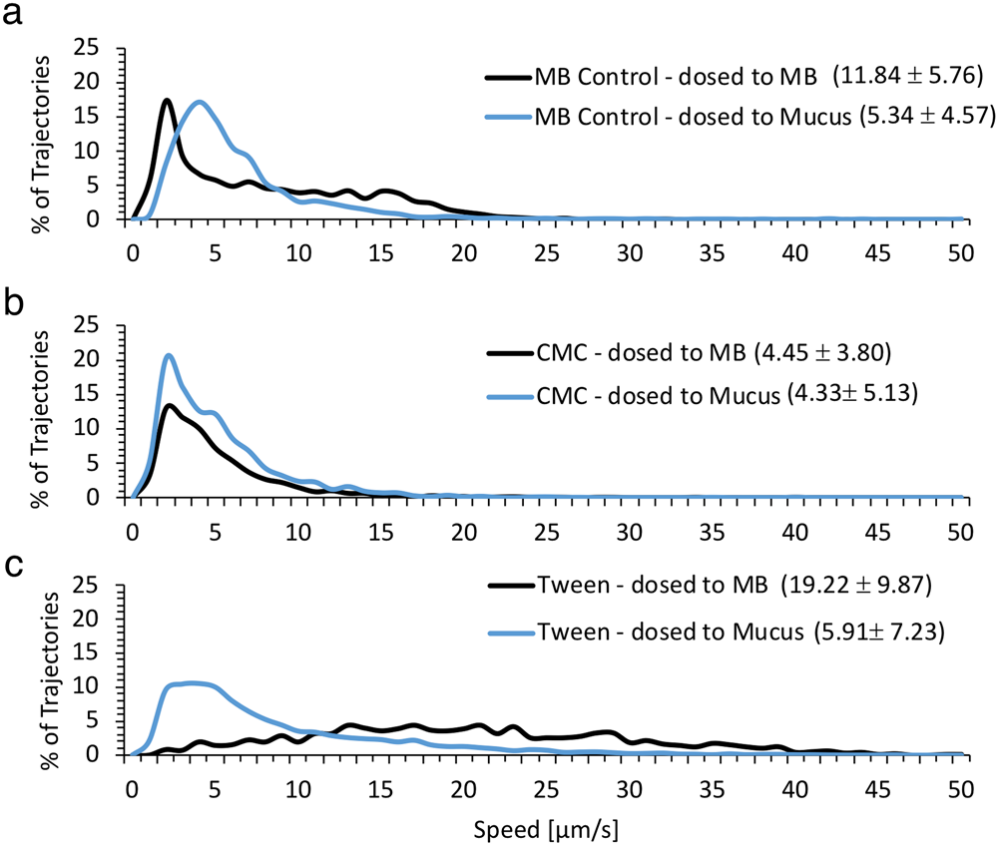
Speed distributions of microbes mixed with MB Control (**a**), CMC (**b**), or Tween (**c**) and dosed to MB or mucus. Microbe median speed ± standard deviation are written next to the figure legend.

Exposure to emulsifiers impacts the mobility of *E*. *coli* both in buffer and within the mucus gel. *E*. *coli* mobility was analyzed in both MB and mucus to explore the impact of CMC, Tween, and mucus on *E*. *coli* (Fig. 6, Supplementary Videos S1–S6). *E*. *coli* speed and the nature of their movement were visually apparent in tracking videos and outlined trajectories. The control groups included *E*. *coli* mixed with MB and dosed to MB or mucus, while test groups included *E*. *coli* mixed with 1% solution of Tween or CMC and dosed to MB or mucus. For the controls, *E*. *coli* had a significantly reduced median speed when dosed to mucus (5.34 ± 4.57 μm/s) relative to MB (11.84 ± 5.76 μm/s) (Fig. 6a). When mixed with CMC, the median *E*. *coli* speed in mucus (4.33 ± 5.13 μm/s) was slightly lower than in MB (4.45 ± 3.80 μm/s) (Fig. 6b). Exposure to CMC significantly reduced *E*. *coli* speed by 18% and 62% when compared to control *E*. *coli* mixed with MB and dosed to mucus and MB, respectively. When mixed with Tween, median *E*. *coli* speed was 5.91 ± 7.23 and 19.22 ± 9.87 μm/s when dosed to mucus and MB, respectively (Fig. 6c). Compared to the control *E*. *coli* dosed to mucus and MB, the presence of Tween increased *E*. *coli* speed by 62% and 10% in MB and mucus, respectively.

### Mucus removal from *in vitro* cell culture

**Figure 7:**
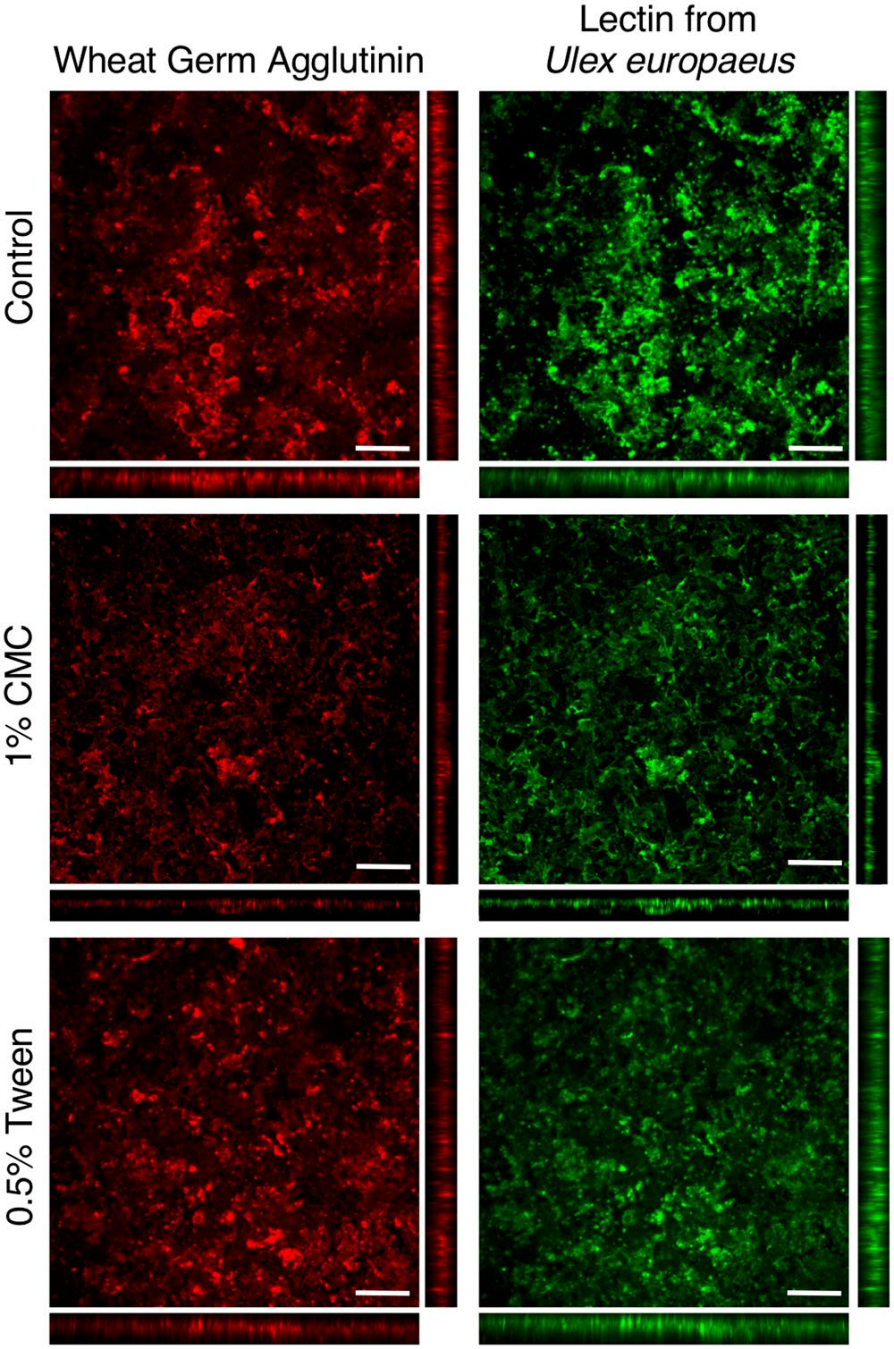
Fluorescence images of Caco-2/HT29-MTX monolayers exposed to MB Control, 1% CMC, or 0.5% Tween and stained with Lectin from *Ulex europaeus* and wheat germ agglutinin. Scale bar: 200 μm. Orthogonal cross-sections are 120 μm thick.

To test if removal of mucus is a mechanism by which exposure to emulsifiers may impact mucosal surfaces, emulsifiers were exposed to mucus-producing intestinal cultures. Emulsifiers resulted in the partial removal of the mucus layer (Fig. 7) and had no impact on Caco-2/HT29-MTX monolayer viability after 1 hr exposure (data not shown). Two different lectins were utilized to visualize the mucus layer: wheat-germ agglutinin (WGA) binds sialic acid and N-acetylglucosaminyl, and lectin from *Ulex europaeus* agglutinin I (UEAI) binds L-fucose. After exposure to emulsifiers, the mucus layer was visibly thinner, and the numbers of globular mucus aggregates were reduced on the cell monolayers compared to control (mean intensity UEAI: 61.97 ± 6.05, WGA: 72.03 ± 6.88). CMC exposure resulted in a decreased degree of staining of both lectins (mean intensity UEAI: 20.40 ± 6.49, WGA: 28.27 ± 6.60) to a greater degree than Tween (mean intensity UEAI: 38.39 ± 8.30, WGA: 52.79 ± 3.28).

### *In vivo* and *ex vivo* intestinal exposure to emulsifiers

**Figure 8:**
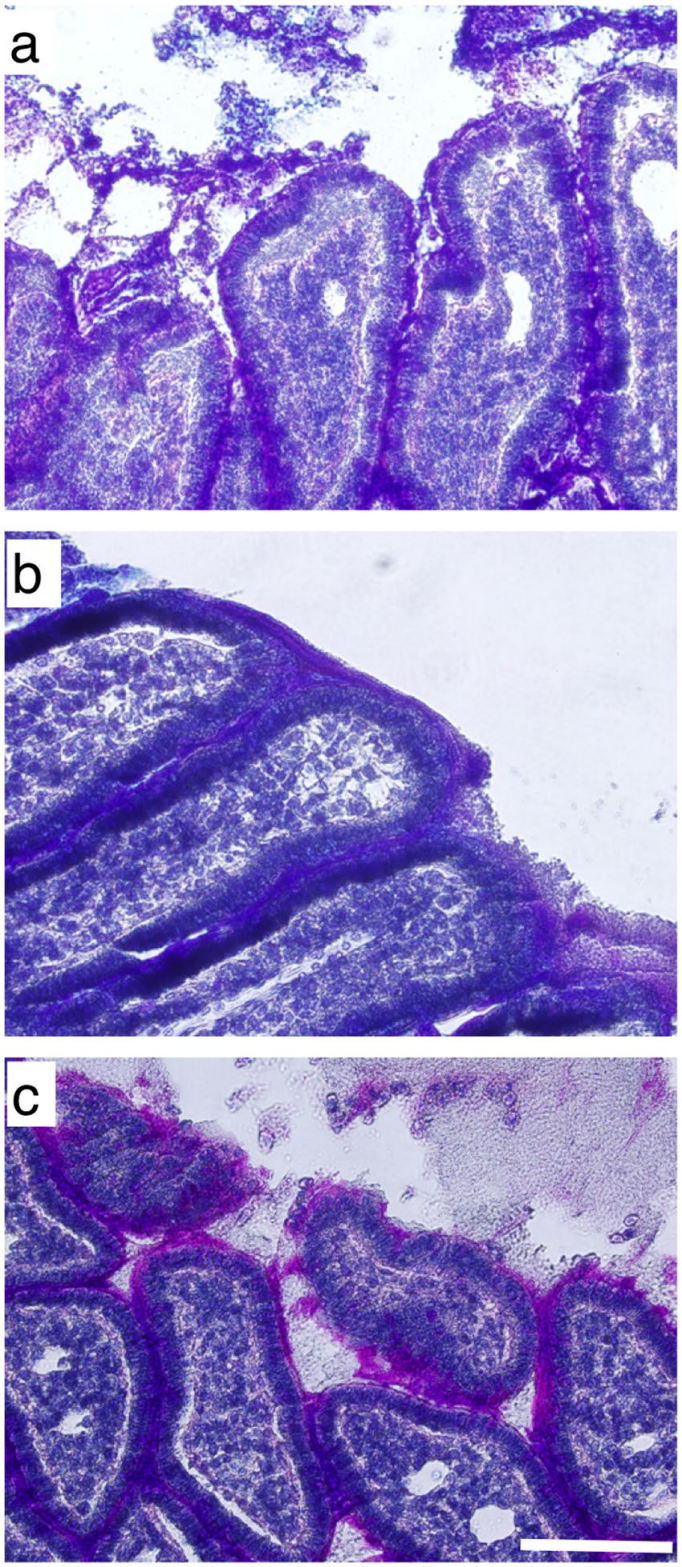
Histological images of rat intestine exposed to to (**a**) MB Control, (**b**) CMC, or (**c**) Tween and stained with Periodic Acid Schiff, alcian blue, and hematoxylin. Scale bar: 100 μm.

Acute exposure to emulsifiers altered the amount and composition of mucus present in rat intestinal loops (Fig. 8, Supplementary Fig. S3). Two stains were used to visualize the mucus layer. Alcian blue stains acidic polysaccharides (e.g., those on mucin) and Periodic Acid Schiff stains carbohydrate containing macromolecules, including both acidic and neutral mucins. A mucus layer that stained positive for neutral and acidic mucins covered the villus tips and was present in the lumen in control (exposed to PBS) intestine. After exposure to CMC, mucus within the lumen was not evident, and the mucus layer was more compacted on the villus tips and stained positive for both neutral and acidic mucins (dark purple in color). In intestine sections exposed to Tween, the mucus layer was looser and had a higher amount of neutral mucins (i.e. stained pink in color) along villus tips compared to MB. Mucus in the lumen was sparse but also had a higher percentage of neutral mucins in the intestine exposed to Tween compared to control.

In an effort to quantify mucus removal due to acute exposure to emulsifiers, rat intestinal tissue was incubated with CMC and Tween in Ussing chambers. Solubilized mucus was quantified using alcian blue assay. Exposure to 1% and 0.5% Tween in Kreb’s buffer significantly enhanced mucus removal relative to Kreb’s buffer control (Supplementary Fig. S4). It was not possible to quantify mucin removed from tissues exposed to CMC since CMC was also pelleted during the centrifugation step in the alcian blue assay. It is possible that alcian blue dye may have been entangled within the CMC polymer network, thus leading to an increase in measured alcian blue concentration, which correlates to mucin concentration. As a result, we do not report the concentration of mucin removed from intestinal tissue after CMC exposure.

## Discussion

**Figure 9:**
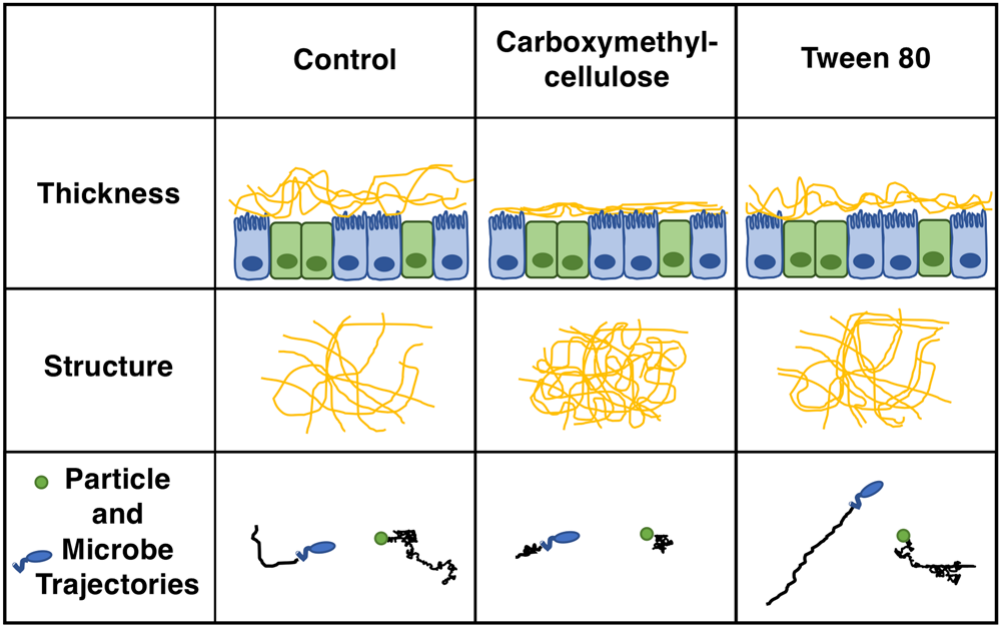
Changes to mucus structural and transport properties after exposure to carboxymethylcellulose and Tween 80.

Recently, the consumption of processed foods and drug formulations containing emulsifiers has increased, and new evidence has suggested a link to altered intestinal microbiota and metabolic syndrome, but the mechanism of action is unclear^16–18,21^. Herein, we report that acute exposure to emulsifiers can directly impact intestinal mucus structure and barrier properties with respect to both passively diffusing (i.e. nanoparticulate) and actively moving (e.g., microbial) entities (Fig. 9, Table 1). As intestinal mucus plays an essential role in controlling interactions of the epithelium and underlying tissues with the microbiota and other lumen contents, the results support the hypothesis that changes in this hydrogel barrier upon acute exposure to emulsifiers may directly contribute to the development of inflammation and metabolic syndrome. These results, together with other reports of changes in mucus secretion and glycosylation in various disease states (i.e. ulcerative colitis, Crohn’s disease, cystic fibrosis)^41–44^ support the overall significance of changes in the mucus barrier in intestinal inflammation and disease.

**Table 1:** Summary of mucus pore size, as well as particle *D*_*eff*_ and microbe speed through mucus exposed to MB Control, CMC, and Tween. Pore size is represented as median ± interquartile range (IQR).

		MB Control	CMC	Tween 80
Pore Size [Median ± IQR]		109.45 ± 112.60	59.3 ± 47.53	88.24 ± 82.28
Particle *D*_*eff*_ [Avg ± STDEV (STDErr)]	Amine	0.014 ± 0.048 (0.00048)	0.00047 ± 0.0029 (0.0000572	0.0131 ± 0.0367 (0.00047)
	Carboxyl	0.042 ± 0.092 (0.00139)	0.0084 ± 0.045 (0.00082)	0.023 ± 0.050 (0.00065)
	PEG	0.067 ± 0.121 (0.0013)	0.017 ± 0.056 (0.00094)	0.046 ± 0.086 (0.0020)
Microbe Speed [Median ± STDEV]	Dosed to MB	11.84 ± 5.76	4.45 ± 3.80	19.22 ± 9.87
	Dosed to Mucus	5.34 ± 4.57	4.33 ± 5.13	5.91 ± 7.23

Particle *D*_*eff*_ is represented as an average ± standard deviation (STDEV) and standard error (STDErr). Microbe speed is represented as median ± STDEV.

Fluorescence and scanning electron microscopy were used to visualize mucin fiber aggregation and changes in pore size after exposure to emulsifiers. Microstructure of mucus exposed to CMC, a mucoadhesive polymer^45,46^, was visualized for the first time with SEM, and reflected considerable modifications to the mucus hydrogel. These structural changes were also evident in fluorescence microscopy images revealing aggregated lectin-stained mucus with defined edges. The change in mucus structure after CMC exposure, i.e. apparent compaction of the mucus gel, may be attributed to the interaction of the CMC polymer chains with mucin fibers. Previously reported rheological analysis of CMC with a 4% (wt/wt) mucin solution support this concept. Experimentally measured bulk viscosity values were found to be higher than theoretical values, which did not consider non-covalent interaction and entanglement between CMC and mucin^46^. Furthermore, the storage modulus (G′) for CMC-mucin relative to CMC-only preparations increased to a greater extent than loss modulus (G″), indicating CMC-mucin interactions imparted a more gel-like structure. The interaction between CMC and mucin can alter the macromolecular organization of the mucus gel, which may have resulted in the observed fluorescent staining pattern of defined mucus edges and decreased pore size observed in SEM micrographs. The interactions between CMC and mucin may also explain the increase in viscoelastic properties of mucus exposed to CMC which was observed from microrheological analysis.

Only in the presence of CMC were amine- and carboxyl- modified particles accumulated along the compacted and defined mucus edges with high lectin staining. Moreover, amine- and carboxyl- modified particles both experienced hindered diffusion, indicating that these charged particles became immobilized in the mucus gel in the presence of CMC. These particle tracking results coupled with fluorescence and SEM images support an altered mucin fiber organization relative to MB, suggesting that charged amine- and carboxyl- modified particles may be associated with CMC-mucin interactions, as charged particles were localized at defined edges in fluorescent images. Conversely, neutral PEG- modified particles were dispersed along edges and in areas with low lectin staining. Particle tracking data showed that PEG- modified particles had <MSD> values that were higher compared to amine- and carboxyl- modified particles in the presence of CMC, but still 4-fold lower compared to dosing in MB. None of the particles were able to penetrate the compacted mucus gel with high lectin staining. We postulate that a decreased pore size inhibited all particle types from diffusing into highly lectin-stained regions.

For a direct investigation of potential implications of mucus structural changes on bacteria translocation, we studied the transport of fluorescently labeled *E*. *coli*. In the presence of CMC, *E*. *coli* mobility was lower compared to controls. This observation was in contrast to a previously published study using a homogeneous solution of 0.05% CMC (MW ~7 × 10^5^), where *E*. *coli* exhibited increased speed and straighter movement compared to buffer alone^47^. In our study, *E*. *coli* were suspended in a solution of 1% CMC for 5 mins and subsequently dosed to MB and allowed to naturally disperse. Thus, this procedure likely resulted in a heterogeneous solution of CMC in MB. A previous study has shown that polymer-polymer interactions are significant in a solution of 1% CMC^47^. In our study, *E*. *coli* may have been entangled within the CMC polymer network, resulting in the observed decrease in speed. When *E*. *coli* were mixed with CMC and dosed to mucus or MB, their speeds were similar. Thus, the reduced speed of *E*. *coli* mixed with CMC and dosed to mucus relative to those mixed with MB and dosed to mucus cannot be directly attributed to mucus structural changes, but rather may be due to a dominant effect of the entangled polymer network. Although we observed a decrease in *E*. *coli* speed in the presence of CMC, fluorescence imaging showed that *E*. *coli* were still present and potentially trapped in small pores with low lectin staining, suggesting that *E*. *coli* may still be able to penetrate a compacted mucus gel.

In agreement with electron and fluorescence microscopy analysis of collected mucus exposed to CMC, *in vivo* animal loop experiments showed that the introduction of CMC resulted in a more compacted mucus layer lining intestinal villi. Moreover, exposure of CMC to a mucus-producing monolayer removed mucus and resulted in a thinner, patchy mucus layer distributed over the cell monolayer. These results suggest that when CMC is ingested, mucus structure may be altered and the gel may be compacted. Outer layers of the compacted mucus coating may be partially removed during peristalsis or food movement, similar to the removal of mucus after pipetting the CMC solution off of the cell cultures. Prior research also showed a reduced mucus layer thickness after chronic, long-term exposure of CMC in drinking water to mice for 12 weeks^16,17^. Moreover, the reduced mucus layer was accompanied by penetration of microbes, including an increase in mucolytic microbes, closer to the epithelium than in controls. Similarly, interleukin 10 (IL-10) gene deficient mice fed 2% CMC over 3 weeks had microbial overgrowth in the mucus layer surrounding small intestine villi, and microbes present in the crypts of Lieberkuehn, in contrast to mice fed water^48^. It is not clear if the reduced mucus layer and ability of microbes to penetrate the underlying epithelium observed after long-term exposure is a secondary effect of inflammation or changes in microbe populations, or a direct effect of exposure to CMC. Our results indicate that direct short-term exposure to CMC can contribute to a structurally altered and thinner mucus barrier. While particle and microbe transport was decreased in the presence of CMC, the altered mucus barrier may allow for closer proximity of both commensal and pathogenic microbes to the epithelium, which may contribute to an inflammatory response.

Minimal mucus structural changes were noted with exposure to Tween. Specifically, there was a slight change in mucus pore size and fluorescent staining pattern compared to MB control. Tween formed micelles at the concentrations tested in this study (critical micelle concentration: 13–15 mg/L), where the average diameter of these micelles in MB was 7.9 ± 0.1 nm, as measured by DLS. Previous work has shown that Tween micelles may interact with lipid depots of pig gastric mucus^49^. Since there was minimal change in mucus microstructure (SEM and fluorescence imaging), we postulate that Tween micelles may diffuse through and interact with hydrophobic regions of the mucus gel, but minimally impact structure.

When fluorescent particles and *E*. *coli* were tracked to study mucus barrier transport properties in the presence of Tween, amine-, carboxyl-, and PEG- modified particles were generally observed throughout the mucus gel, similar to MB control. Amine- modified particles dosed to mucus were not affected by Tween, potentially due to electrostatic interactions with mucin fibers being the dominating force controlling their motion. However, the diffusion of both carboxyl- and PEG- modified particles decreased in Tween treated samples relative to MB. Another non-ionic detergent, (nonylphenoxy)polyethylene oxide, significantly decreased the diffusion of 200 nm PEG-modified particles (160-fold) and mucus pore size^50^. These changes were attributed to the disruption of hydrophobic interactions within mucus resulting in the dispersion of mucin fibers. We hypothesize that Tween also alters hydrophobic interactions within the mucus gel; however, the effect was not as significant as that observed with (nonylphenoxy)polyethylene oxide, as the presence of Tween resulted in only a 5-fold decrease in PEG- modified particle diffusion and minimal changes to the mucus pore size. Moreover, microrheological analysis indicated that elastic and storage moduli were slightly increased for Tween exposed mucus samples compared with MB Control indicating a more solid-like gel, which may also explain the decrease in carboxyl- and PEG- modified particle diffusion. The presence of Tween increased *E*. *coli* speed both in MB and in mucus. Although the complex mucus gel hindered *E*. *coli* transport, *E*. *coli* dosed to mucus in the presence of Tween had a faster speed compared to *E*. *coli* in MB dosed to mucus. Prior research similarly showed that movement of *E*. *coli* RP437 on motility assay plates was increased in the presence of Tween compared to control^51^. In another study, microbe adhesion to polystyrene decreased when suspended in media with a lower surface tension^52,53^. Likewise in our studies, Tween, a hydrophilic non-ionic surfactant, may have reduced the interfacial surface tension within the mucus gel, resulting in increased motility of *E*. *coli*. Tween has also been shown to alter cell membrane microviscosity^54^, which may have contributed to the increased translocation of *E*. *coli* isolated from Crohn’s disease patients across microfold cell monolayers compared to control (not exposed to Tween)^55^. These results suggest that Tween can increase microbial translocation across the mucus and epithelial layer, which may lead to the progression of intestinal inflammation.

After intestinal loops were exposed to Tween, mucus covering villi appeared similar in thickness to controls, and mucus was present in the intestinal lumen. The Tween-exposed loops had similar neutral mucin staining compared to control loops, however, there was a decrease in the amount of negatively-charged mucin sugars. Similar to intestinal loops, cell monolayers exposed to Tween had a larger decrease in the amount of negatively-charged sugars relative to neutral sugars compared with control. The loss of these mucin sugars is important, as they may impact bacterial binding and translocation to the epithelium^7^. Specifically, sialic acid and sulfate residues give mucin molecules their strong negative charge. These highly negatively-charged mucins are reported to bind pathogenic and commensal microbes, and protect the epithelium from bacterial enzymatic degradation^7,56^. The loss of these negatively-charged mucins combined with faster microbial mobility observed in this study upon Tween exposure could allow for microbes to penetrate the mucus layer and bind to the epithelium. Ussing chamber and cell culture studies indicated that Tween depletes the mucus layer. Previous work using a rat jejunum single pass *in situ* perfusion model further supported the loss of mucus, where the administration of 1% Tween increased the concentration of mucus and lactate dehydrogenase in the perfusate compared with saline^57^. We hypothesize that the disruption of hydrophobic regions and the slight change in mucus structure in the presence of Tween may allow for the dispersion of mucin fibers, thus contributing to the removal of mucus.

## Conclusions

In summary, the intestinal mucus barrier plays an important role in regulating the diffusion of nutrients and the proximity of microbes and foreign particulates to the epithelium. The impact of food-associated stimuli, i.e. emulsifiers (CMC and Tween), on mucus structure and barrier properties with respect to both passively diffusing entities (nanoparticles) and *E*. *coli* was investigated. Results demonstrated significant acute changes to mucus gel structure, thickness, and composition upon exposure to emulsifiers, as well as particle diffusion and *E*. *coli* transport in mucus. Specifically, CMC decreased mucus gel pore size and thickness as visualized in SEM micrographs, as well as in *in vivo* intestinal loops and *in vitro* cell culture experiments. Moreover, both particle and *E*. *coli* transport were significantly inhibited in CMC exposed mucus compared with control. Tween had minimal effects on *ex vivo* porcine mucus structure, but resulted in the depletion of the mucus layer in *in vitro* cell culture and *ex vivo* tissue experiments, changes in mucus content after exposure *in vivo*, and increased *E*. *coli* speed in mucus. These results thus suggest that oral exposure to CMC may result in compaction of intestinal mucus, and some associated loss of clumped material, while exposure to Tween may result in selective loss of mucosal components and altered microbial binding and transport. The acute impact of emulsifiers on mucus amount, structure, and barrier properties may contribute to the exposure of the epithelium to intestinal lumen contents and increase in bacterial translocation, resulting in the progression of intestinal inflammation. Future studies further exploring mechanisms by which mucus changes lead to onset of inflammation *in vivo* could motivate rational design of prophylactic or therapeutic measures to address intestinal inflammation.

## Electronic supplementary material

Video S1

Video S2

Video S3

Video S4

Video S5

Video S6

Supplementary Information Document
