# Supplementary material for: Acute Exposure to Commonly Ingested Emulsifiers Alters Intestinal Mucus Structure and Transport Properties
Source: Sci Rep. 2018 Jul 3;8:10008. doi: 10.1038/s41598-018-27957-2 (PMC6030187; doi:10.1038/s41598-018-27957-2)
Supplement: Supplementary file 7 — Supplementary Information Document [file 41598_2018_27957_MOESM7_ESM.docx]

Acute Exposure to Commonly Ingested Emulsifiers Alters Intestinal Mucus Structure and Transport Properties

Jaclyn Y. Lock^†^, Taylor Carlson^‡^, Chia-Ming Wang^†^, Albert Chen^‡^, Rebecca L. Carrier^†‡*^

^†^Department of Bioengineering, ^‡^Department of Chemical Engineering, Northeastern University, Boston, Massachusetts 02115 USA

*corresponding author. Email: Rebecca@coe.neu.edu

**Supplementary Information:**

c

b

a

**Supplementary Figure S1**: Scanning electron micrographs of mucus exposed overnight to MB Control (a), CMC (b), or Tween (c). Scale bar: 2 μm.

**Supplementary Figure S2**: Macroscopic images of mucus at t = 0, 1min, and 2hrs after addition of particle solution containing Amine (A), Carboxyl (C), and PEG (P) particles in MB Control, CMC, or Tween.

MB

CMC

Tween

**Supplementary Figure S3**: Histological images of different rat intestines exposed to to MB Control, CMC, or Tween and stained with Periodic Acid Schiff, alcian blue, and hematoxylin. Scale bar: 100 μm.

To quantify mucus removal by Tween, Ussing chambers were utilized. Animal experiments were approved by Northeasteran IACUC (protocol #15-0316R). Briefly, male Wistar rats, 7-8 weeks old, 200-250 g in weight, were fasted ~16 hrs overnight on a wire-bottom cage to prevent coprophagy. Rats were euthanized with carbon dioxide, the small intestine was removed and ~2 cm of ileum was cut-open lengthwise to expose the mucosal surface. The tissue was loaded into the Ussing chamber. The reservoir facing the mucosal side was loaded with Krebs bicarbonate Ringer (KBR) buffer, 0.5% Tween dissolved in KBR, or 1% Tween dissolved in KBR, while the reservoir facing the serosal side was loaded with KBR. A gas mixture of 95% oxygen and 5% carbon dioxide was gently bubbled in both reservoirs. At 5, 10, 20, 30, 40, 50, and 60 mins, 300 μL of liquid from the mucosal reservoir was removed and replaced with fresh KBR, 0.5% Tween in KBR, or 1% Tween in KBR. Mucin concentration was quantified using the alcian blue assay. Specifically, 100 μL of sample was loaded into a 96 well plate and 33.4 μL of alcian blue (Sigma Aldrich) was added. The plate was equilibrated on a shaker for 2hrs at room temperature and then centrifuged for 30 mins at 1870 g. the plate was washed twice with wash buffer (40% ethanol, 0.1 M sodium acetate, 25 mM magneiusm chloride, at pH 5.8). The pellet was then resuspended in 100 μL of 10% sodium dodecyl sulfate. Sample absorbance was measured at 620 nm. Results indicated that exposure to Tween removed mucin significantly more compared to buffer alone. Moreover, mucin removal was concentration dependent, such that 1% Tween resulted in more mucus removal compared to 0.5% Tween.

Mucin Concentration [µg/mL]

| Time [mins] | Kreb's Buffer | 0.5% Tween | 1% Tween |
| --- | --- | --- | --- |
| 5 | 1.23807 | 9.28169 | 30.4062 |
| 10 | 2.63254 | 12.4081 | 33.7266 |
| 20 | 5.54688 | 32.0615 | 36.6172 |
| 30 | 4.04404 | 34.3108 | 41.8906 |
| 40 | 3.01856 | 30.6403 | 50.9922 |
| 50 | 14.6514 | 40.4333 | 44.3867 |
| 60 | 9.27288 | 39.4363 | 39.0781 |

**Supplementary Figure S4**: Mucin removed from rat intestine Ussing experiment when exposed to MB Control, 0.5% and 1% Tween for 1 hr. Error bars represent standard deviation. 0.5% Tween and 1% Tween were both statistically different compared with Kreb’s buffer with p < 0.05 at all time-points.

**Video S1**: 20 s video of microbes mixed with MB and dosed to MB.

**Video S2**: 20 s video of microbes mixed with MB and dosed to mucus.

**Video S3**: 20 s video of microbes mixed with CMC and dosed to MB.

**Video S4**: 20 s video of microbes mixed with CMC and dosed to mucus.

**Video S5**: 20 s video of microbes mixed with Tween and dosed to MB.

**Video S6**: 20 s video of microbes mixed with Tween and dosed to mucus.
